# Early‐Onset Bilateral Clear Cell Renal Cell Carcinoma in a Pediatric Renal Transplant Recipient: A Rare Case Report

**DOI:** 10.1155/criu/7275534

**Published:** 2026-04-10

**Authors:** Ravi Kiran Gautam, Mahesh Bahadur Adhikari, Ajit Khadga, Bipin Maharjan, Pramesh Prasad Shrestha, Prashant Mishra, Deepak Kumar Yadav, Birodh Basnet, Subodh Ghimire, Arnab Singh Parmar

**Affiliations:** ^1^ Department of Urology and Renal Transplant, Nepal Mediciti, Lalitpur, Nepal; ^2^ Department of Pathology, Nepal Mediciti, Lalitpur, Nepal

**Keywords:** acquired cystic kidney disease, bilateral RCC, clear cell carcinoma, pediatric transplant, renal cell carcinoma, renal transplantation

## Abstract

**Background:**

Renal transplantation (RTx) is the preferred treatment for end‐stage renal disease (ESRD), reducing mortality and improving quality of life. However, long‐term immunosuppression increases the risk of malignancy, with renal cell carcinoma (RCC) occurring in approximately 0.6%–0.7% of renal transplant recipients, most commonly arising in the native kidneys. Bilateral RCC is rare and is typically associated with papillary histology and acquired cystic kidney disease (ACKD).

**Case Presentation:**

A 16‐year‐old male with ESRD secondary to diffuse mesangial hypercellularity underwent living‐related renal transplantation from his mother. Fourteen months posttransplant, routine ultrasonography revealed bilateral renal masses in the native kidneys. Contrast‐enhanced computed tomography confirmed multiple lesions in both kidneys. The patient underwent bilateral laparoscopic nephrectomy. Histopathology revealed bilateral clear cell renal cell carcinoma, confined to the kidneys with negative surgical margins. Multiple benign cortical cysts suggested early acquired cystic kidney disease. At 1‐year follow‐up, the patient showed no evidence of recurrence, with normal serum creatinine and stable graft function.

**Discussion:**

Bilateral RCC after renal transplantation is rare and is usually associated with papillary histology and acquired cystic kidney disease. The early occurrence of bilateral clear cell RCC in a pediatric transplant recipient is unusual and may indicate an underlying predisposition. Early detection through routine imaging allowed timely surgical management with preservation of graft function.

**Conclusion:**

This case highlights the rare occurrence of early bilateral clear cell RCC in a pediatric renal transplant recipient. Careful surveillance of native kidneys in transplant recipients may facilitate early diagnosis and favorable outcomes.

## 1. Introduction

The history of organ transplantation dates back to skin autografting in India during the sixth century BC [1]. The first successful renal transplant, a landmark event in transplant medicine, was performed on December 23, 1954, by Dr. Joseph Murray in Boston between identical twin brothers, ushering in a new era of organ transplantation [2].

Renal transplantation (RTx) remains the preferred renal replacement therapy for most patients with end‐stage renal disease (ESRD), reducing long‐term mortality by 48%–82% compared with patients remaining on dialysis while awaiting transplantation and significantly improving quality of life [2, 3]. In Nepal, RTx was successfully established on August 8, 2008, at Tribhuvan University Teaching Hospital (TUTH), Kathmandu, following the legalization of live donor transplantation in 2000, with Bir Hospital becoming the second transplant center in December 2008 [4, 5]. Nepali law restricts organ donation to close relatives to prevent organ trade [4].

Despite its benefits, renal transplant recipients face an approximately twofold increased risk of malignancy due to chronic immunosuppression, and malignancy remains a major cause of mortality along with infections and cardiovascular disease [6]. Posttransplant renal cell carcinoma (RCC) is an important concern and most commonly arises in the native kidneys, although it may rarely occur in the renal allograft. The development of RCC in transplant recipients has been associated with prolonged immunosuppression, dialysis exposure, and conditions such as acquired cystic kidney disease (ACKD) [6, 7].

In this report, we present the rare case of a 16‐year‐old male who developed bilateral RCC in the native kidneys 1 year after RTx, highlighting the clinical presentation, pathological findings, management, and implications for surveillance in pediatric renal transplant recipients.

## 2. Case Presentation

A 16‐year‐old male with ESRD secondary to diffuse mesangial hypercellularity (DMH) had a peak serum creatinine of 17.8 mg/dL prior to initiation of maintenance hemodialysis (MHD). He underwent MHD twice weekly for 1 month before optimization for RTx. Human leukocyte antigen (HLA) typing revealed a 5/6 match with his mother, who served as the living‐related donor. The patient subsequently underwent a successful living‐related renal transplantation (LRRTx).

Fourteen months posttransplant, he remained on regular follow‐up without any new complaints. However, ultrasonography of the abdomen revealed renal masses in both native kidneys. Contrast‐enhanced computed tomography (CECT) demonstrated that the native right kidney measured 7.8 × 3.2 cm and the native left kidney measured 8.0 × 3.0 cm. Both kidneys showed multiple simple cysts without evidence of calculus or hydronephrosis.

In the right native kidney, masses measuring 23.8 × 23.3 mm in the upper pole and 17.1 × 17.2 mm in the lower pole were identified. In the left native kidney, multiple lesions were noted: in the interpolar region (lateral aspect) measuring 16.2 × 15.0, 9.4 × 8.4, and 4.6 × 4.6 mm; in the interpolar region (medial aspect) measuring 11.0 × 10.9 mm; and in the lower pole measuring 4.0 × 3.0 mm (Figure 1a,b).

Figure 1(a, b) Coronal and sagittal CT views of bilateral native kidneys showing renal masses. (a) Coronal view showing the left kidney with multiple lesions (up to 16.2 × 15.0 mm) and the right kidney with two tumors (1.8 and 1.5 cm), along with simple cysts. (b) Sagittal view demonstrating the graft kidney and confirming bilateral renal masses with cysts, without calculus or hydronephrosis.

**Figure:**
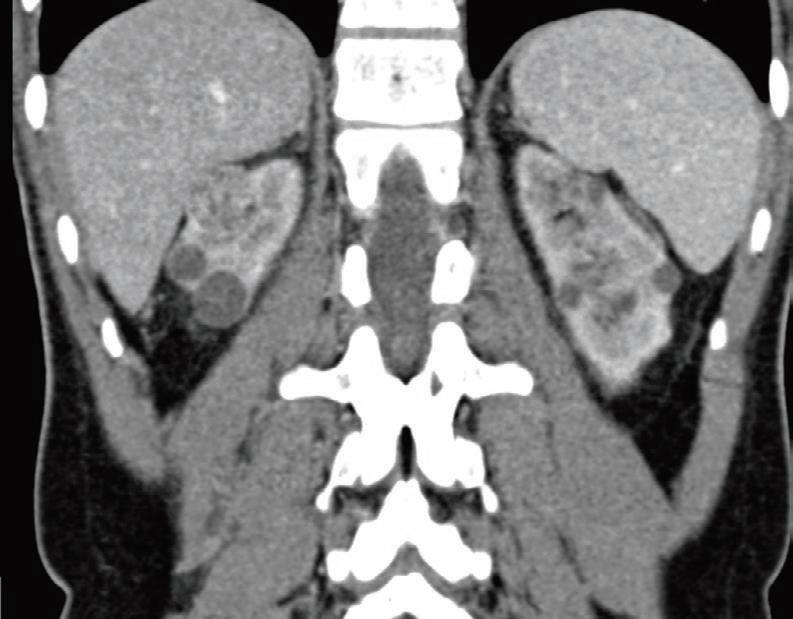
(a)

**Figure:**
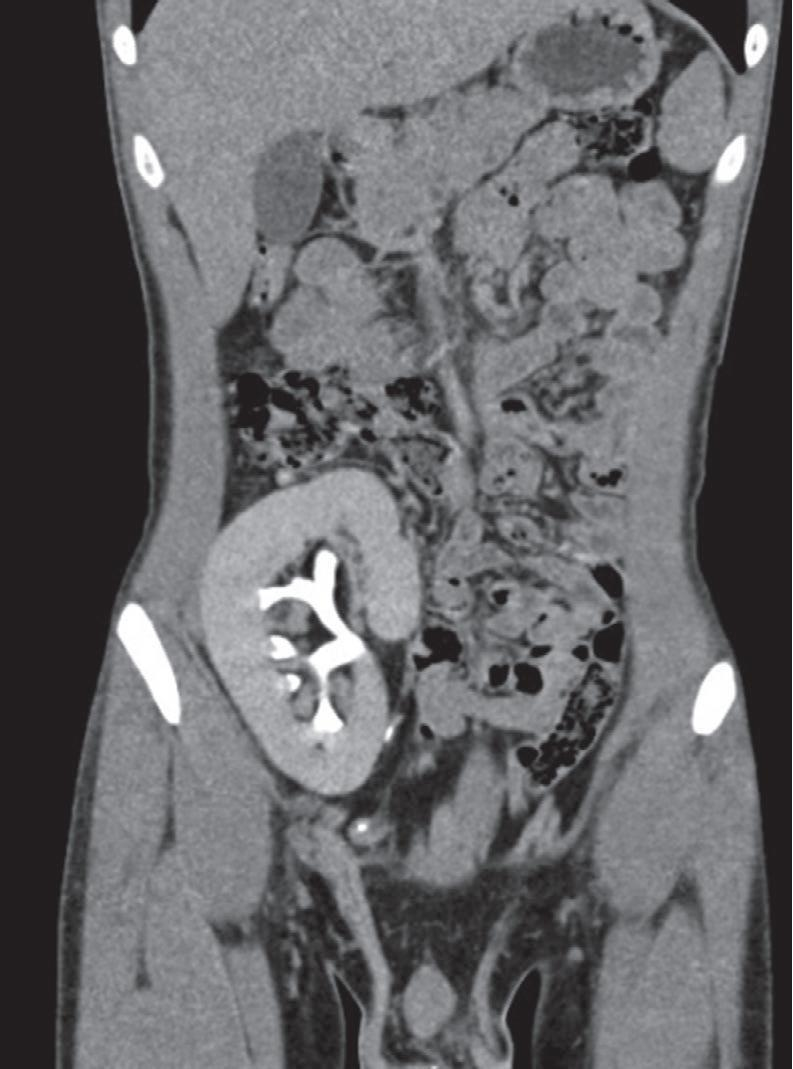
(b)

Considering the absence of established management guidelines for such cases, a detailed literature review was performed and bilateral laparoscopic nephrectomy was planned. The procedure was performed under general anesthesia with endotracheal intubation. The patient was initially placed in the right lateral decubitus position for left laparoscopic nephrectomy, followed by the left lateral decubitus position for right nephrectomy, and finally in the supine position for specimen retrieval. A total of four ports were used, including one camera port and one epigastric port that served as common access points for both sides (Figure 2a,b). Specimen retrieval was performed through a midline incision (Figure 3a,b).

Figure 2(a, b) Port placement and patient positioning for bilateral laparoscopic nephrectomy. (a) Patient positioning for right nephrectomy in the left lateral decubitus position. (b) Patient positioning for left nephrectomy in the right lateral decubitus position.

**Figure:**
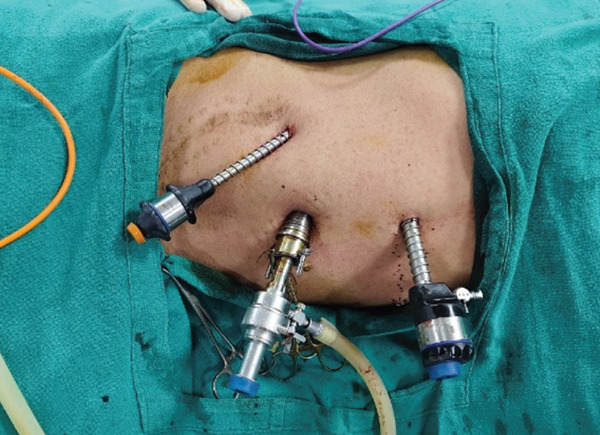
(a)

**Figure:**
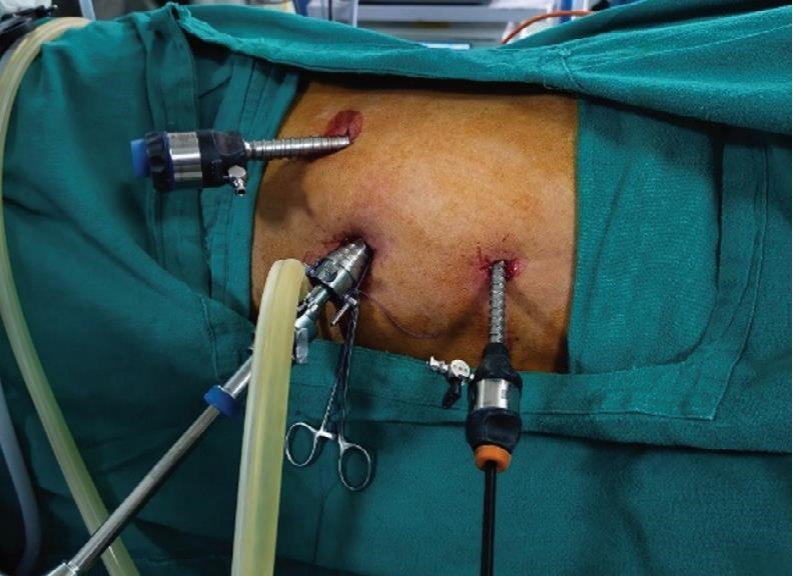
(b)

Figure 3(a, b) Specimen retrieval following bilateral laparoscopic nephrectomy. (a) Intraoperative image showing specimen removal through a midline incision. (b) Gross appearance of the bilateral kidney specimens.

**Figure:**
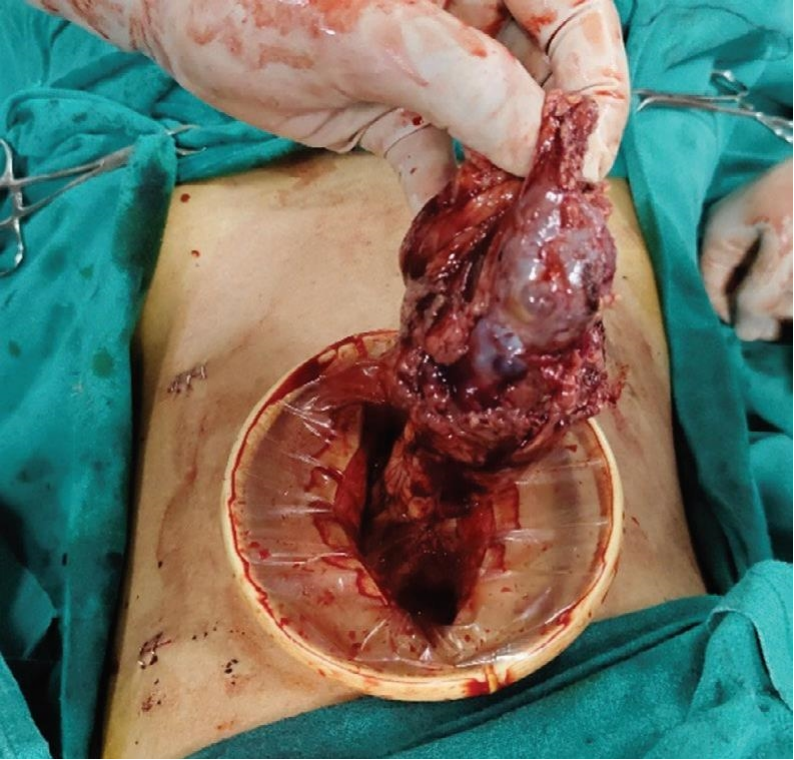
(a)

**Figure:**
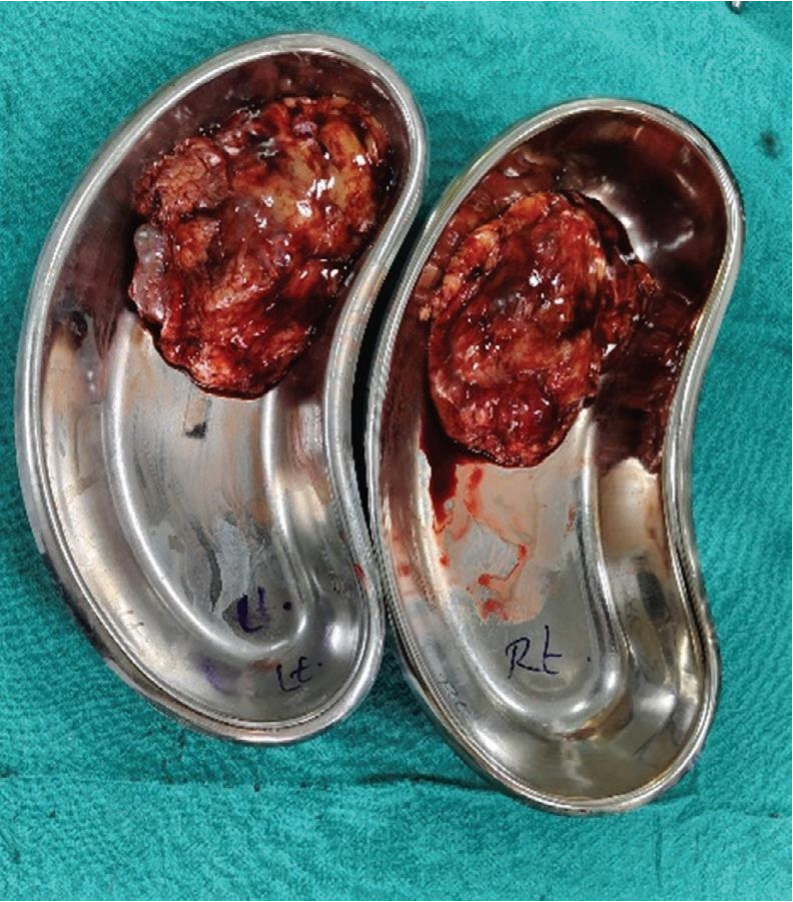
(b)

On gross examination, the right kidney measured 9.0 × 4.0 × 2.0 cm with a 0.5 cm attached ureter, no suprarenal gland, and an intact capsule. The cut surface revealed two tumors: a 1.8 cm exophytic yellowish‐brown variegated solid tumor and a 1.5 cm grey‐white to grey‐yellow tumor. Multiple uniloculated cortical cysts measuring 0.5–1.5 cm with thin walls (0.1–0.2 cm) containing clear fluid were also present. The renal sinus, hilar vessels, and perinephric fat were sampled (Figure 4a).

Figure 4(a, b) Gross specimen and cut section of the right and left native kidneys.

**Figure:**
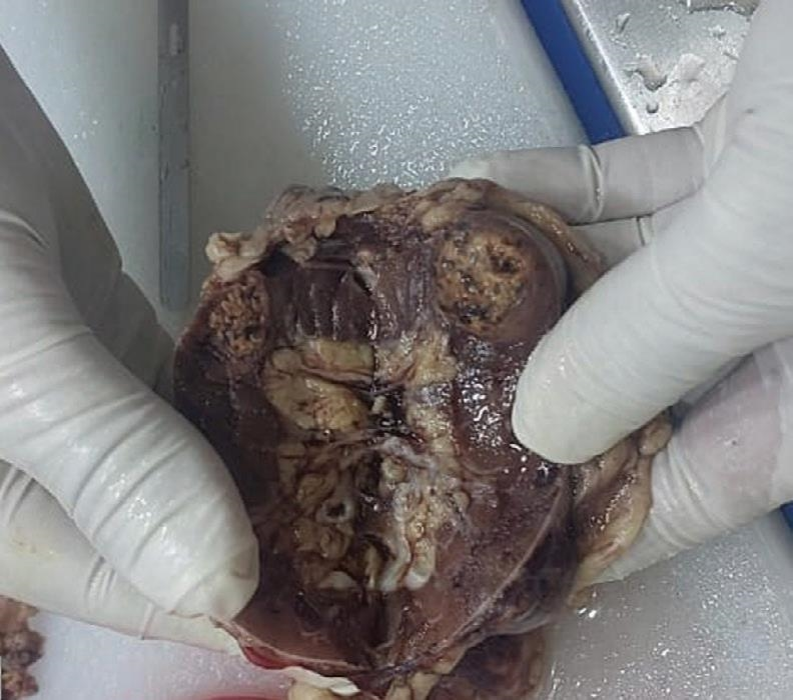
(a) Right

**Figure:**
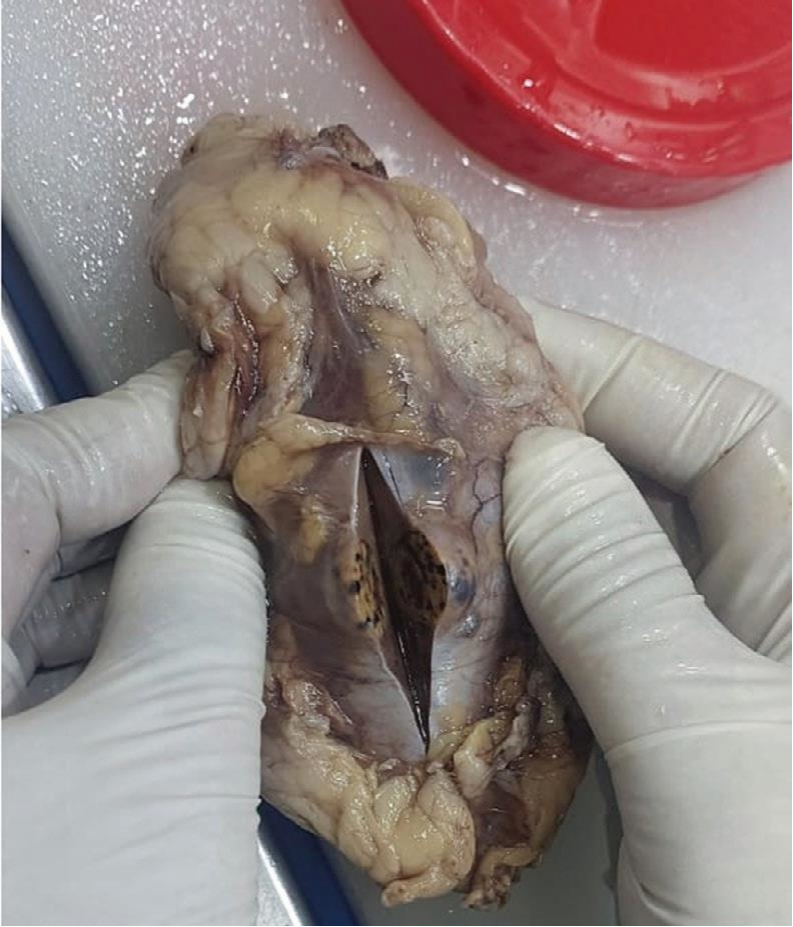
(b) Left

The left kidney measured 10.0 × 5.0 × 1.5 cm with a 2 cm attached ureter, no suprarenal gland, and an intact capsule. The cut surface showed a 1.0 cm exophytic yellowish‐brown variegated solid tumor in the mid pole along with multiple cortical cysts measuring 0.5–1.5 cm with thin walls (0.2–0.3 cm) containing clear fluid (Figure 4b).

Microscopic examination of both kidneys revealed clear cell renal cell carcinoma (ccRCC), WHO/ISUP Grade 1. Tumor cells demonstrated inconspicuous nucleoli at 400× magnification. The left kidney contained a unifocal 1.0 cm tumor in the mid pole staged as pT1a pN0, whereas the right kidney showed multifocal tumors measuring 1.8 cm and 1.5 cm staged as mpT1a pN0. All tumors were confined to the kidney without sarcomatoid or rhabdoid features, tumor necrosis, or lymphovascular invasion. Surgical margins were negative. One reactive lymph node was identified in the perinephric fat of the left kidney, whereas no lymph nodes were found in the right kidney. Multiple benign renal cortical cysts lined by thin nonneoplastic epithelium were also present (Figure 5a,b).

Figure 5(a, b) Microscopic view of bilateral clear cell renal cell carcinoma.

**Figure:**
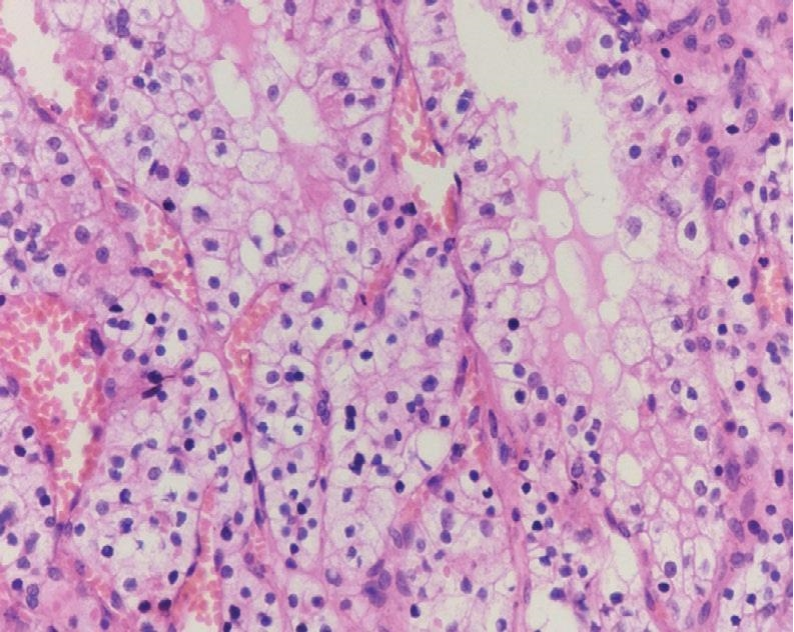
(a) Right clear cell RCC

**Figure:**
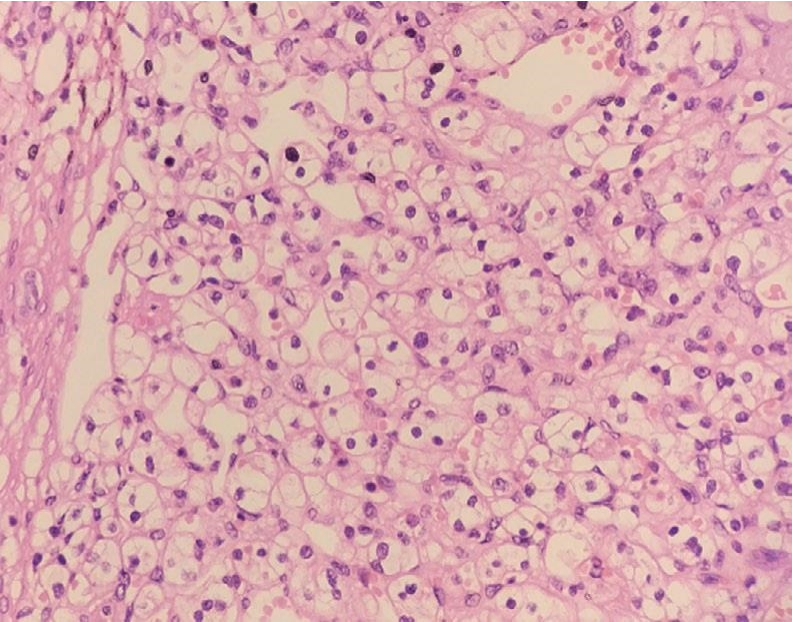
(b) Left clear cell RCC

At the 1‐year follow‐up, contrast‐enhanced CT showed no evidence of local recurrence, metastasis, or new lesions. Serum creatinine remained within the normal range and the patient maintained stable graft function, reflecting an excellent clinical outcome for bilateral ccRCC.

## 3. Discussion

RCC following RTx is a recognized complication, with an overall reported incidence of approximately 0.6%–0.7%. These tumors occur more frequently in the native kidneys (0.7%) than in the renal allograft (0.2%) [8, 9]. The increased risk has been attributed to multiple factors, including chronic immunosuppression, ACKD, prolonged dialysis duration, and patient‐related risk factors such as older age, male sex, and specific underlying causes of ESRD [6, 10]. Bilateral RCC arising in native kidneys is exceptionally rare, particularly among pediatric transplant recipients, and most reported cases have demonstrated papillary histology [11]. In contrast, the present case describes a 16‐year‐old male who developed bilateral clear cell RCC diagnosed 1 year after RTx, representing an unusual presentation compared with the predominantly papillary histology reported in previously described bilateral cases (Table 1).

**Table 1:** Reported cases of bilateral renal cell carcinoma after renal transplantation.

First author (year), country	Sex	Age (*y*)	Years after transplantation	Immunosuppressive drug	Tumor size (cm) (L/R)	RCC type	Presenting signs	Stage (L/R)	Surgery	Follow‐up	Outcome
Carmellini 1995 [12], Italy	Male	45	4.8	Cyclosporin + prednisone	5.0/5.0	Granular cell carcinoma	Intermittent fever	T3	Bilateral nephrectomy with lymphadenectomy	6 months	Died of RCC
Okumi 2001 [13], Japan	Male	47	10.1	NA	1.6/3.3	Alveolar and granular subtype	No	T1a	Bilateral nephrectomy	30 months	AWRM
Ianhez 2007 [14], Brazil	NA	NA	NA	NA	3.0	Papillary	NA	NA	Bilateral nephrectomy	4 months	AWRM
Nakashita 2008 [15], Japan	Female	26.5	8.5	Methylprednisolone + cyclosporin	4.0/4.5	Clear cell	Microscopic hematuria	NA	Staged bilateral laparoscopic nephrectomy	18 months	AWRM
Klatté 2009 [16], Italy	Male	58.7	3.3	NA	2.5/1.6	Papillary	No	T1a	NA	5.1 years	Died of other cause
Bing 2011 [17], United States	Male	38.6	15.3	NA	1.5/1.4	Papillary	No	T1a	NA	4.6 years	AWRM
	Male	46.7	11.6	NA	2.0/6.5	Papillary	No	T1a/T1b	NA	6.7 years	AWRM
	Male	45.9	14.1	NA	0.7/1.5	Clear cell	No	T2a	NA	8.3 years	AWRM
	Female	67	2	Tacrolimus + mycophenolate + prednisone	1.1/2.2	Clear cell papillary	NA	T1a	Bilateral nephrectomy	21 months	AWRM
Cheung 2011 [18], China	Male	48	12.3	Cyclosporin + prednisone	1.5/6.0	Papillary	NA	T1a/T1b	Bilateral nephrectomy	119 months	AWRM

Abbreviations: AWRM, alive without recurrence or metastasis; L, left; NA, not available; R, right; RCC, renal cell carcinoma.

The pathogenesis of post‐RTx RCC is closely tied to ACKD, which develops in 5%–20% of patients initiating dialysis and nearly all after prolonged dialysis exposure, significantly increasing the risk of renal malignancy [7–19]. Pediatric studies suggest that ACKD may occur in approximately 21.6%–45.8% of children receiving dialysis, with longer dialysis duration being a major contributing factor [20]. More recent data further demonstrate that ACKD may be present in up to 42% of children undergoing kidney replacement therapy, with dialysis duration ≥ 28 months significantly associated with its development [21]. Although successful RTx may reduce cyst size, chronic immunosuppression promotes oncogenesis by impairing immune surveillance mechanisms [22]. In addition, ACKD is recognized as a precursor for a distinct tumor subtype known as acquired cystic disease–associated renal cell carcinoma (ACD‐RCC), which has been included as a separate entity in the World Health Organization classification of renal tumors [23]. Unlike the more common papillary RCC observed in CKD‐related bilateral cases, the clear cell histology in this patient resembles sporadic RCC but remains low‐grade and organ‐confined, contributing to a favorable prognosis [24]. Systematic reviews indicate a decreasing RCC incidence over time, possibly due to improved pre‐RTx screening, yet mortality remains unchanged at approximately 13.9%, highlighting the need for vigilant surveillance [8, 9].

Screening guidelines for post‐RTx RCC vary. The European Renal Best Practice and European Association of Urology recommend annual ultrasound screening for high‐risk groups such as patients with ACKD or a history of RCC, whereas Kidney Disease Improving Global Outcomes and the American Society of Transplantation do not endorse universal screening due to insufficient evidence demonstrating a mortality benefit [25, 26]. In the present case, incidental detection during routine follow‐up aligns with previous reports describing RCC discovered during imaging performed for erythrocytosis, hematuria, or rising serum creatinine levels [27]. The early detection and favorable outcome in this patient suggest that routine ultrasonography during the early posttransplant period—particularly in pediatric patients or those at risk of developing ACKD—may facilitate early diagnosis and treatment. However, the cost‐effectiveness and optimal screening interval for such surveillance strategies require further investigation [28].

Bilateral RCC in native kidneys after RTx is rare and is most often reported in adults several years post‐RTx, typically with papillary histology. Surgical removal of the native kidneys generally provides favorable outcomes for localized tumors [29, 30]. In contrast, the present case is notable for the patient′s young age and clear cell histology. These findings raise the possibility of an underlying hereditary predisposition, such as von Hippel–Lindau syndrome, although genetic testing was not performed. Bilateral nephrectomy achieved complete tumor removal while preserving allograft function, consistent with current recommendations for the management of localized native kidney RCC in transplant recipients [30].

## 4. Conclusion

This case underscores the rarity of early bilateral clear cell RCC post‐RTx in a pediatric patient, contrasting with the predominantly papillary histology in reported bilateral cases. Prompt surgical intervention and enhanced surveillance are critical for favorable outcomes in this high‐risk population.

## Author Contributions

A.K.: conceptualization, data collection, manuscript drafting, and manuscript guarantor. All authors: manuscript review.

## Funding

No funding was received for this manuscript.

## Disclosure

All authors have read and approved the final version of the manuscript. Ajit Khadga had full access to all of the data in this study and takes complete responsibility for the integrity of the data and the accuracy of the data analysis.

## Consent

Written informed consent for publication of this case report and accompanying images was obtained from the patient and his guardian.

## Conflicts of Interest

The authors declare no conflicts of interest.

## Data Availability

The data that support the findings of this study are available on request from the corresponding author. The data are not publicly available due to privacy or ethical restrictions.
